# Emotional health concerns of oncology physicians in the United States: Fallout during the COVID-19 pandemic

**DOI:** 10.1371/journal.pone.0242767

**Published:** 2020-11-24

**Authors:** Lauren Thomaier, Deanna Teoh, Patricia Jewett, Heather Beckwith, Helen Parsons, Jianling Yuan, Anne H. Blaes, Emil Lou, Jane Yuet Ching Hui, Rachel I. Vogel

**Affiliations:** 1 Division of Gynecologic Oncology, University of Minnesota, Minneapolis, MN, United States of America; 2 Division of Hematology, Oncology and Transplantation, University of Minnesota, Minneapolis, MN, United States of America; 3 Division of Health Policy and Management, University of Minnesota, Minneapolis, MN, United States of America; 4 Department of Radiation Oncology, University of Minnesota, Minneapolis, MN, United States of America; 5 Department of Surgery, University of Minnesota, Minneapolis, MN, United States of America; Chiba Daigaku, JAPAN

## Abstract

**Introduction:**

Cancer care is significantly impacted by the Coronavirus Disease 2019 (COVID-19) pandemic. Our objective was to evaluate the early effects of the pandemic on the emotional well-being of oncology providers across the United States and explore factors associated with anxiety and depression symptoms.

**Materials and methods:**

A cross-sectional survey was administered to United States cancer-care physicians recruited over a two-week period (3/27/2020–4/10/2020) using snowball-convenience sampling through social media. Symptoms of anxiety and depression were measured using the Patient Health Questionnaire (PHQ-4).

**Results:**

Of 486 participants, 374 (77.0%) completed the PHQ-4: median age was 43 years; 63.2% female; all oncologic specialties were represented. The rates of anxiety and depression symptoms were 62.0% and 23.5%, respectively. Demographic factors associated with anxiety included female sex, younger age, and less time in clinical practice. Perception of inadequate personal protective equipment (68.6% vs. 57.4%, p = 0.03) and practicing in a state with more COVID-19 cases (65.8% vs. 51.1%, p = 0.01) were associated with anxiety symptoms. Factors significantly associated with both anxiety and depression included the degree to which COVID-19 has interfered with the ability to provide treatment to cancer patients and concern that patients will not receive the level of care needed for non-COVID-19 illness (all p-values <0.01).

**Conclusion:**

The perceived degree of interference with clinical practice along with personal concerns about COVID-19 were significantly associated with both anxiety and depression among oncology physicians in the United States during the COVID-19 pandemic. Our findings highlight factors associated with and sources of psychological distress to be addressed to protect the well-being of oncology physicians.

## Introduction

By early April 2020, the Coronavirus Disease 2019 (COVID-19) pandemic resulted in over 600,000 cases and over 24,500 deaths in the United States [1]. Its burden on the healthcare system cannot be overstated. The pandemic has led to drastic changes in the delivery of health care: the creation of additional surge capacity for COVID-19 patients, and in oncology specifically, changes in work schedules and roles, rapid implementation of telehealth, delays of procedures, treatments and ambulatory visits, suspension of clinical trials, changes in end-of-life care, and implementation of policies on the usage of personal protective equipment (PPE) [2]. Additionally, while cancer is considered a potential risk factor for COVID-19, it is not yet clear to what extent cancer increases the risk of being infected with COVID-19, or of developing complication in its disease course, though studies are starting to be published [3–5]. These many changes and uncertainties in oncology practice affect individuals with cancer and their treating physicians. However, other than a few editorials or commentaries [6, 7], no research to date has focused on the emotional well-being of physicians delivering cancer care in the United States.

While increased emotional strain and post-traumatic stress symptoms have been reported in COVID-19 frontline healthcare workers [8–10], potential effects of the pandemic on other providers, such as oncologists, have received less attention. The drastic change in oncology practice due to COVID-19 and the uncertainty of its impact on the outcome of vulnerable cancer populations may cause significant stress among oncologists. We sought to evaluate the early effects of the COVID-19 pandemic on the emotional health of oncology physicians across the United States and explore factors associated with anxiety and depression symptoms.

## Materials and methods

We conducted a cross-sectional anonymous online survey among physicians who treat individuals with cancer in the United States [11]. The study was reviewed and approved by the University of Minnesota Institutional Review Board. Eligibility criteria included: ≥18 years old, ability to read/write in English, and being a physician (MD or DO) currently residing and providing cancer treatment to patients in the United States.

Individuals were recruited over a two-week period (March 27, 2020 –April 10, 2020) using snowball convenience sampling through social media (Twitter, Facebook, LinkedIn). Survey items included demographics, measures of clinical practice, concerns about COVID-19, effects of the pandemic on treatment decision-making and practice, and emotional well-being, using REDCap for data collection and storage [12]. All questions were optional. We used the Patient Health Questionnaire (PHQ-4), a brief validated self-report screener to measure symptoms of depression and anxiety over the past two weeks [13]. Potentially clinically relevant anxiety and depression symptoms were calculated using an established cut-off score of ≥3 [14, 15]. The number of COVID-19 cases in each state was ascertained from the Centers of Disease Control and Prevention (CDC) as of April 3, 2020, the half-way point during the study recruitment period [1] and the number of cases per 100,000 residents was calculated using United States Census Bureau 2019 population estimates.

Participant characteristics and responses were summarized using descriptive statistics, reporting medians, ranges, and absolute and relative frequencies as appropriate. We explored the associations of demographic, clinical practice, and COVID-19 related factors with self-reported depression and anxiety by comparing those with and without symptoms using Chi-squared tests and Fisher’s Exact tests as appropriate for categorical variables, and Wilcoxon rank sum tests for continuous variables. As a sensitivity analysis, we used multivariable logistic regression models to further examine the relationship between elevated symptoms of anxiety or depression and COVID-related concerns, adjusted for age, gender, race and the number of COVID cases in state of practice. P-values <0.05 were considered statistically significant. Data were analyzed using SAS 9.4 (Cary, NC).

## Results

A total of 548 individuals started the survey; 62 were screened out as not eligible (32 not physicians who offer cancer treatment to patients, 30 not practicing in the United States). Of 486 eligible participants, 374 (77%) completed the PHQ-4 questions (primary outcome) and therefore were included in this analysis. Those who completed the PHQ-4 questions were similar to those who did not with the exception that those who reported practicing at an academic institution were less likely to complete those questions. Approximately two-thirds (63.2%) of respondents were female and the median age was 43 years (range 31–78 years; Table 1). Just over half of respondents reported having minor children living with them, with females reporting this only slightly more often than males (61.8% vs. 54.1%, p = 0.34). Respondents practiced across 43 states with all oncologic specialties represented. Physicians reported treating a wide range of cancer types; the most common cancers treated were breast (58.5%), colon (38.8%), melanoma (32.1%), rectal (31.3%) and pancreatic (29.9%). More than half practiced in an academic setting, in a large city or its suburb, and at large or medium sized hospitals.

**Table 1:** Demographic and clinical practice characteristics (N = 374).

Characteristic	N	Median (Range)
**Age, years**	337	43 (31–78)
**Time in practice, years**	357	10 (0.5–45)
	**N**	**%**
**Gender**		
Male	133	35.8
Female	235	63.2
Non-binary gender identification	4	1.1
*Missing*	*2*	
**Race/Ethnicity**		
White, non-Hispanic	272	75.1
Asian Indian	38	10.5
Chinese	16	4.4
Hispanic	12	3.3
Other	24	6.6
*Missing*	*12*	
**Children under 18 living with you**		
No	151	40.8
Yes	219	59.2
*Missing*	*4*	
**Medical Specialty**		
Surgeon	211	56.6
Medical Oncology	89	23.9
Radiation Oncology	54	14.5
Other	19	5.1
*Missing*	*1*	
**Practice at an academic institution**		
No	171	46.1
Yes	200	53.9
*Missing*	*3*	
**Hospital Size**		
Small hospital (fewer than 100 beds)	30	8.1
Medium hospital (100–499 beds)	160	43.0
Large hospital (500 or more beds)	170	45.7
Ambulatory clinic only (no inpatients)	12	3.2
*Missing*	*2*	
**Type of community (practice)**		
Rural area	18	4.9
Small city or town	81	21.8
Suburb near a large city	95	25.6
Large city	177	47.7
*Missing*	*3*	

Most (74.8%) respondents were practicing in states with at least 1,000 confirmed COVID-19 cases at the time of the survey (Table 2). The majority (60.1%) reported being moderately or extremely concerned about getting COVID-19 and 20.3% considered themselves to be at high-risk for developing serious illness from COVID-19. Physicians reported COVID-19 had affected their ability to provide treatment to cancer patients to a moderately severe degree as determined by a visual analogue scale with 0 indicating “no problem” and 100 indicating “severe problem” (median score 72; severity range 0–100). Radiation oncologists reported lower interference than surgeons and medical oncologists (p = 0.003). A majority were moderately or extremely concerned about transmitting COVID-19 to a patient (65.9%) and the inability for their patients to receive the adequate level of care for a severe illness other than COVID-19 (70.5%). Surgeons reported less concern about giving their patients COVID than medical or radiation oncologists (p = 0.002).

**Table 2:** COVID-19 exposure and concerns (N = 374).

Characteristic	N	%
**Number of COVID-19 cases in practicing state (as of 04/03/2020)**		
101–500	13	3.7
501–1000	77	21.6
1001–5000	133	37.4
5001 or more	133	37.4
*Missing (did not provide state where practice)*	*18*	
**Number of COVID-19 cases in practicing state (as of 04/03/2020), per 100,000 residents (estimates 2019)**		
0–19	120	33.7
20–49	153	43.0
50–99	42	11.8
100+	41	11.5
*Missing (did not provide state of practice)*	*18*	
**Do you consider yourself to be at “high risk” for severe illness from COVID-19?**		
No	257	68.7
Yes	76	20.3
Unsure	41	11.0
*Missing*	*0*	
**How concerned are you about getting COVID-19?**		
Not at all concerned/Slightly concerned/Somewhat concerned	149	40.0
Moderately concerned/Extremely concerned	224	60.0
*Missing*	*1*	
**How concerned are you about one of your family members getting COVID-19 from you?**		
Not at all concerned/Slightly concerned/Somewhat concerned	78	20.9
Moderately concerned/Extremely concerned	279	74.6
Already happened	2	0.5
Not applicable	15	4.0
*Missing*	*0*	
**How concerned are you about your patients getting COVID-19 from you?**		
Not at all concerned/Slightly concerned/Somewhat concerned	126	34.2
Moderately concerned/Extremely concerned	243	65.9
*Missing*	*5*	
**Adequate PPE for clinical practice**		
No	156	41.9
Yes	216	58.1
*Missing*	*2*	
**How concerned are you about your patients getting the level of healthcare they need if they become extremely ill from something other than COVID-19?**		
Not at all concerned/Slightly concerned/Somewhat concerned	110	29.5
Moderately concerned/Extremely concerned	263	70.5
*Missing*	*1*	
	**N**	**Median (Range)**
**To what degree has COVID-19 interfered with your ability to provide treatment to active cancer patients? (0 = no problem, 100 = severe problem)**	337	72 (0–100)

Almost two-thirds (62.0%) of oncology physicians in this study reported anxiety symptoms (Table 3). Demographic characteristics associated with anxiety included gender identification as female, younger age, and fewer years in clinical practice. Further, many items related to perceived COVID-19 risks were associated with anxiety, including practicing in a state with >1000 COVID-19 cases (65.58% vs. 51.1%, p = 0.01) or having inadequate PPE access (68.6% vs. 57.4%, p = 0.03). Almost one-quarter (23.5%) of respondents reported depression symptoms. While approximately a third who reported anxiety symptoms also reported depression symptoms (36.6%), almost all who reported depression symptoms were also anxious (96.6%). Asian-Indian physicians were more likely to report depression (36.8%) than non-Hispanic White (23.9%) or other race/ethnicity physicians (13.5%; p = 0.04). Both anxiety and depression were associated with being moderately or extremely concerned about getting COVID-19, transmitting it to a family member or a patient, or inability for a patient to access an adequate level of care for a serious non-COVID-19 related illness (all p<0.01). The associations of COVID-19 related concerns with elevated symptoms of anxiety or depression remained after adjusting for age, gender, race and number of COVID cases in state of practice (S1 Table).

**Table 3:** Associations between demographic, clinical practice and COVID-19 variables and anxiety and depression symptoms (N = 374).

	Anxiety*					Depression*				
	No		Yes			No		Yes		
	N = 142 (38.0%)		N = 232 (62.0%)			N = 286 (76.5%)		N = 88 (23.5%)		
Characteristic	N	Median (Range)	N	Median (Range)	p-value	N	Median (Range)	N	Median (Range)	p-value
**Age, years**	125	45	212	42	**0.0448**	256	43	81	42	0.3829
		(31–75)		(31–78)			(31–75)		(32–78)	
**Time in practice, years**	136	12	221	10	**0.0244**	272	11	85	9	0.2785
		(1–45)		(0.5–40)			(0.5–45)		(0.5–40)	
**COVID-19 interfered with ability to provide treatment to active cancer patients?**	128	68	209	75	**0.0007**	259	70	78	74.5	0.0753
		(0–100)		(0–100)			(0–100)		(0–100)	
(0 = no problem, 100 = severe problem)										
	**N**	**%**	**N**	**%**	**p-value**	**N**	**%**	**N**	**%**	**p-value**
**Gender**					**<0.0001**					0.3157
Male	71	53.4	62	46.6		107	80.5	26	19.6	
Female	67	28.5	168	71.5		174	74.0	61	26.0	
Non-binary gender identification	2	50.0	2	50.0		3	75.0	1	25.0	
**Race**					0.2839					**0.0362**
White, non-Hispanic	102	37.5	170	62.5		207	76.1	65	23.9	
Asian Indian	10	26.3	28	73.7		24	63.2	14	36.8	
Other	22	42.3	30	57.7		45	86.5	7	13.5	
**Children under 18 living with you**					0.1343					0.2222
No	64	42.4	87	57.6		120	79.5	31	20.5	
Yes	76	34.7	143	65.3		162	74.0	57	26.0	
**Medical Specialty**					0.5276					0.8392
Surgeon	81	38.4	130	61.6		159	75.4	52	24.6	
Medical Oncology	32	36.0	57	64.0		68	76.4	21	23.6	
Radiation Oncology	24	44.4	30	55.6		44	81.5	10	18.5	
Other	5	26.3	14	73.7		15	79.0	4	21.1	
**Hospital Size**					0.1944					0.7060
Small hospital (fewer than 100 beds)	12	40.0	18	60.0		23	76.7	7	23.3	
Medium hospital (100–499 beds)	68	42.5	92	57.5		126	78.8	34	21.3	
Large hospital (500 or more beds)	58	34.1	112	65.9		125	73.5	45	26.5	
Ambulatory clinic only (no inpatients)	2	16.7	10	83.3		10	83.3	2	16.7	
**Type of community (practice)**					0.6016					0.5703
Rural area	5	27.8	13	72.2		13	72.2	5	27.8	
Small city or town	33	40.7	48	59.3		64	79.0	17	21.0	
Suburb near a large city	39	41.1	56	59.0		76	80.0	19	20.0	
Large city	63	35.6	114	64.4		130	73.5	47	26.6	
**Practice at an academic institution**					0.3366					0.4759
No	69	40.4	102	59.7		128	74.9	43	25.2	
Yes	71	35.5	129	64.5		156	78.0	44	22.0	
COVID-19 cases in practicing state**					**0.0131**					0.3527
1000 or less	44	48.9	46	51.1		72	80.0	18	20.0	
1001 or more	91	34.2	175	65.8		200	75.2	66	24.8	
COVID-19 cases in practicing state**, **per 100,000 residents**					0.5226					0.6400
<50	106	38.8	167	61.2		207	75.8	66	24.2	
50+	29	34.9	54	65.1		65	78.3	18	21.7	
**Do you consider yourself to be at “high risk” for severe illness from COVID-19?**					0.2931					0.5763
No	104	40.5	153	59.5		200	77.8	57	22.2	
Yes	26	34.2	50	65.8		57	75.0	19	25.0	
Unsure	12	29.3	29	70.7		29	70.7	12	29.3	
**Concern about getting COVID-19**					**<0.0001**					**0.0055**
Not at all/Slightly/Somewhat concerned	88	59.1	61	40.9		125	83.9	24	16.1	
Moderately/Extremely concerned	53	23.7	171	76.3		160	71.4	64	28.6	
**Concern about family members getting COVID-19 from you**					**<0.0001**					**0.0043**
Not at all/Slightly/Somewhat concerned	50	64.1	28	35.9		70	89.7	8	10.3	
Moderately/Extremely concerned	89	31.9	190	68.1		208	74.6	71	25.5	
**Concern about your patients getting COVID-19 from you**					**0.0022**					**0.0071**
Not at all/Slightly/Somewhat concerned	61	48.4	65	51.6		107	84.9	19	15.1	
Moderately/Extremely concerned	78	32.1	165	67.9		176	72.4	67	27.6	
**Adequate PPE for clinical practice**					**0.0283**					0.1709
No	49	31.4	107	68.6		114	73.1	42	26.9	
Yes	92	42.6	124	57.4		171	79.2	45	20.8	
**Concern about your patients getting the level of healthcare they need if they become extremely ill from something other than COVID-19**					**<0.0001**					**0.0095**
Not at all/Slightly/Somewhat concerned	59	53.6	51	46.4		94	85.5	16	14.6	
Moderately/Extremely concerned	83	31.6	180	68.4		192	73.0	71	27.0	

*Established cut-off for identifying potentially clinically relevant anxiety and/or depression using the PHQ-4

**Centers for Disease Control, as of 04/03/2020

## Discussion

Oncology physicians report significant anxiety and depression symptoms early in the COVID-19 pandemic in the United States. Importantly, the perceived degree of interference with clinical practice along with personal concerns about COVID-19 were significantly associated with both anxiety and depression.

Previous studies from Wuhan, China have shown evidence of the high prevalence of distress, anxiety, and depression in COVID-19 “frontline” personnel, including nurses and physicians [8, 9] Risk factors for emotional health symptoms included female sex, nursing profession, working on the “frontline,” and working in Wuhan, China [9]. While it is not difficult to explain the pandemic’s effects on the mental health of frontline healthcare workers, it is equally important to understand how the physician-based workforce and hospital care capabilities for other specialties have been affected. Oncology physicians have historically been more susceptible to burnout [16], and could be at even higher risk following a crisis such as COVID-19. Wu et al. recently published a comparison of 190 oncology physicians and nurses who were either working in oncology or dispatched to the “frontline” [10]. Those continuing to work in their usual capacity with uninfected cancer patients surprisingly had a greater frequency of burnout symptoms. Our study did not measure all aspects and symptoms that define physician burnout, but our findings of high anxiety and depression among oncologists and the association of these symptoms with concerns about COVID-19 and patients getting adequate care for non-COVID-19 conditions are generally consistent with that study.

Strengths of this study include the national sample of oncologists treating a wide range of cancers at a time the pandemic was evolving in the United States. Limitations include its survey-based design, social media recruitment, and high proportions of surgeons and females; these respondents may not fully represent all oncologists practicing in the United States. We also chose to focus on physicians as they are heavily involved in treatment decision-making for oncology patients, however, we would expect similar results for advanced practice providers in oncology settings as well. These data may also under-report emotional symptoms due to social desirability bias. Finally, we do not know the rates of anxiety and depression among these oncologists prior to the pandemic and cannot infer these high rates were directly caused by the pandemic. However, the prevalence of anxiety and depression in our study, 62.0% and 23.5%, respectively, are higher than what has been previously reported. In a systematic review and meta-analysis performed in 2017 evaluating psychiatric distress and morbidity specifically among oncologists, the authors reported 27% experienced psychiatric morbidity and 12% screened positive for depression [17]. A 2015 review suggested that one third of the general population will experience an anxiety disorder over their lifetime [18], however, data are very limited on anxiety symptoms among oncologists; one study in Brazil reported rates of 19% in 2018 [19].

The prevalence of anxiety-related symptoms we observed among oncology physicians in the United States is alarming. These findings support a recent call to action to address emotional distress among physicians even prior to the pandemic [20]. It is crucial to protect the mental health of all oncologists in order to preserve their ability to deliver high quality and efficient care to cancer patients at a time of unprecedented uncertainty. Further studies are needed to identify the sources of psychological distress and assess the efficacy of interventions for physicians during and after the COVID-19 pandemic. We have made tremendous strides in cancer care over the past few decades and the risk of losing oncologist capacity to depression and anxiety would be an extra insult to the already tragic situation of cancer and COVID-19.

## Supporting information

S1 Table Multivariable logistic regression models assessing the associations between COVID-19 related concerns and anxiety and depression symptoms (N = 374). (DOCX)
