# Supplementary material for: Emotional health concerns of oncology physicians in the United States: Fallout during the COVID-19 pandemic
Source: PLoS One. 2020 Nov 24;15(11):e0242767. doi: 10.1371/journal.pone.0242767 (PMC7685431; doi:10.1371/journal.pone.0242767)
Supplement: S1 Table — (DOCX) [file pone.0242767.s001.docx]

**S1 Table. Multivariable logistic regression models assessing the associations between COVID-19 related concerns and anxiety and depression symptoms (N=374).**

|  | **Anxiety*** | | **Depression*** | |
| --- | --- | --- | --- | --- |
| **Characteristic** | **Odds Ratio (95% CI)**** | **P-value** | **Odds Ratio (95% CI)**** | **P-value** |
| **Concern about getting COVID-19** |  | <0.0001 |  | 0.0268 |
| Not at all concerned/Slightly concerned/Somewhat concerned | 1.00 |  | 1.00 |  |
| Moderately concerned/Extremely concerned | 4.71 (2.81, 7.91) |  | 1.95 (1.08, 3.50) |  |
| **Concern about family members getting COVID-19 from you** |  | <0.0001 |  | 0.0317 |
| Not at all concerned/Slightly concerned/Somewhat concerned | 1.00 |  | 1.00 |  |
| Moderately concerned/Extremely concerned | 4.47 (2.38, 8.42) |  | 3.51 (1.33, 9.28) |  |
| **Concern about your patients getting COVID-19 from you** |  | 0.0019 |  | 0.0052 |
| Not at all concerned/Slightly concerned/Somewhat concerned | 1.00 |  | 1.00 |  |
| Moderately concerned/Extremely concerned | 2.30 (1.36, 3.90) |  | 2.58 (1.33, 5.02) |  |
| **Adequate PPE for clinical practice** |  | 0.0260 |  | 0.0540 |
| No | 1.77 (1.07, 2.92) |  | 1.69 (0.99, 2.87) |  |
| Yes | 1.00 |  | 1.00 |  |
| **Concern about your patients getting the level of healthcare they need if they become extremely ill from something other than COVID-19** |  | 0.0002 |  | 0.0083 |
| Not at all concerned/Slightly concerned/Somewhat concerned | 1.00 |  | 1.00 |  |
| Moderately concerned/Extremely concerned | 2.70 (1.60, 4.53) |  | 2.44 (1.26, 4.75) |  |

**Established cut-off for identifying potentially clinically relevant anxiety and/or depression using the PHQ-4*

*** Adjusted for age, gender, race and number of COVID cases in state*
